# Margin status impact on recurrence of phyllodes tumors in high-risk groups: a retrospective observational study

**DOI:** 10.1186/s12885-023-11805-2

**Published:** 2024-01-09

**Authors:** Aliyeh Ranjbar, Vahid Zangouri, Mansoureh Shokripour

**Affiliations:** 1 Breast Diseases Research Center, Shiraz University of Medical Sciences, Shiraz, Iran; 2 Surgical Oncology Division, General Surgery Department, Shiraz University of Medical Sciences, Shiraz, Iran; 3 Department of Pathology, Medical School, Shiraz University of Medical Sciences, Shiraz, Iran

**Keywords:** Phyllodes tumour, Local recurrence, Surgical margins, Risk factor

## Abstract

**Background:**

Phyllodes tumor (PT) is an fibroepithelial tumor with potential for local recurrence. The optimal margin for surgical resection of PT is still debated, particularly in cases of positive margins. This study aimed to identify the risk factors for phyllodes tumor recurrence and the effect of a free margin on tumor recurrence by considering these risk factors.

**Materials and methods:**

This is a retrospective observational study of patients diagnosed with PT who had undergone surgical management. The data were collected from medical records from 2001 to 2020 in the breast clinic of Shahid Motahhari Clinic of Shiraz. Patients were followed up for at least 3 years after the operation to be checked for local recurrence or distant metastasis at regular intervals.

**Results:**

This retrospective study included 319 patients with PT who underwent surgical management. Of these patients, 83.9% (*n* = 267), 7.6% (*n* = 24), and 8.5% (*n* = 27) were classified as benign, borderline, and malignant, respectively. 8.8% of all patients and 7.6% of non-malignant cases experienced local recurrence, and risk factors for recurrence included oral contraceptive use, smoking, size > 4 cm, stromal overgrowth, and stromal cell atypia. A negative surgical margin decreased the prevalence of recurrence in tumors > 4 cm and with stromal overgrowth significantly.

**Conclusion:**

The study found that a negative margin in all patients did not reduce the recurrence rate in benign and borderline phyllodes tumors, suggesting close follow up as a reasonable alternative. However, a negative margin may be effective in reducing recurrence in certain high-risk groups.

## Introduction

Phyllodes tumors (PT) is a rare fibroepithelial breast tumor with an incidence ranging from 0.3 to 0.9%, primarily affecting women aged between 35 and 55 years [1–3]. It originates from the periductal stromal cells of the breast and is histologically characterized by increased stromal cellularity with leafy fronds [4]. The World Health Organization classifies this tumor into benign, borderline, or malignant based on its histological features [5], with the benign tumor being the most common grade, occurring in 60–75% of cases [6].

PT is often clinically and behaviorally similar to fibroadenomas of the breast, which are the most common benign breast tumors [4, 7]. Mammography and breast ultrasound cannot distinguish phyllodes tumors from fibroadenomas [7–9], and fine-needle biopsy is often insufficient for diagnosis [7, 10–12]. Consequently, phyllodes tumor is often diagnosed after excision, as it has poor preoperative diagnostic accuracy [13]. Historically, Phyllodes tumors have been known to have a high potential for local recurrence [13–16]. Traditionally, surgical margins were considered as the most important predictor of local recurrence, and a free margin of at least 1 cm was recommended to reduce recurrence [17–19]. However, multiple cohort studies have shown the lack of association between surgical margin width and local recurrence rates, questioning the need for negative margins in benign phyllodes tumors [20–23]. Although the National Comprehensive Cancer Network (NCCN) practice guidelines endorse surgical excision without obtaining surgical margins for benign phyllodes tumors [24]. However, wide-local excision with margins > 1 cm remains a standard practice in borderline and malignant PT [24].

Currently, the optimal margin for surgical resection of PT is still a topic of debate. In many medical centers, the standard practice for the management of close or positive margins in PT is to perform wide local excision and re-excision [25, 26]. There is also evidence suggesting that achieving a clear surgical margin during the initial surgery can reduce the risk of tumor recurrence [27]. This study aims to identify the risk factors for phyllodes tumor recurrence and the effect of a free margin on tumor recurrence by considering these risk factors.

## Method

### Patient and study design

This is a retrospective observational study of patients diagnosed with phyllodes tumors who had undergone surgical management. All patients referred to the breast clinic of Shahid Motahhari Clinic in Shiraz between 2001 and 2020 with a final diagnosis of phyllodes tumor was included in the study. The inclusion criteria were age between 18 and 80 years and a final diagnosis of phyllodes tumor. The exclusion criteria were a simultaneous diagnosis of other breast malignancies and serious medical illness that prevents the patients’ follow-up.

It is important to note that patients with malignant PT received adjuvant radiotherapy after surgery, while those with benign and borderline PT did not receive any additional treatment except surgery. Specifically, all patients with malignant PT received adjuvant radiotherapy for 15 sessions, 5 days per week, after surgery.

### Data collection

Data were collected from medical records from 2001 to 2020 in the breast clinic of Shahid Motahhari Clinic of Shiraz. The data included age, past medical history, mammographic and ultrasonographic tumor characteristics, type of surgery (local excision, wide excision, mastectomy, re-excision after initial surgery), tumor size, and margin according to pathology reports. Slides were reviewed by a single expert pathologist to review the margin status and determine histological features such as histologic subtype (according to WHO classification), heterogeneous stroma, stromal overgrowth defined as stromal proliferation without epithelial elements observed in at least one low-power field (×4 microscope objective), stromal cell atypia, and mitotic rate. In this study, “Negative margin” means no tumor on ink.

### Patient follow-up

All participants were checked for local recurrence (new tumor found in the ipsilateral breast) or distant metastasis at least 3 years after the operation. They were followed up at intervals of 4 months in the first year with ultrasonography and breast examination, and every 6 months in the second year. The duration of follow-up was annually. Additionally, mammography was done annually for patients over 35 years of age.

### Statistical analysis

The statistical analysis was done using the Package for the Social Sciences (SPSS) 21 software. Parametric data are presented as mean ± standard deviation. Categorical values were compared using the chi-square or Fisher’s exact chi-square tests, and Student’s t-test was used for the comparison of continuous variables. Recurrence rates and recurrence-free probabilities were estimated using the Kaplan-Meier method. The association of variables with recurrence was evaluated using Cox proportional-hazard regression analysis and summarized with HR and 95% CI. Univariate and multivariate analysis models were used, and only factors found to be associated with recurrence in the univariate analysis were entered into the multivariate model. A p-value < 0.05 was considered statistically significant.

### Ethics approval

This study was conducted in accordance with the principles established by the Declaration of Helsinki and obtained the approval of the Ethics Committee of Shiraz University of Medical Sciences (approval ID: IR.SUMS.MED.REC.1400.029).

## Result

Over the study period, 341 patients with a diagnosis of PT had undergone breast surgery. A total of 23 patients were excluded from the study, with 18 excluded due to incomplete medical records or follow-up and five due to a diagnosis of breast cancer during the follow-up period. This study comprised 319 patients with a mean age of 36.97 years and a mean follow-up period of 114.97 months.

22% (*n* = 70) of the lesions were sampled by Core needle biopsy (CNB). The most common pathological diagnosis on biopsy was a fibroepithelial lesion, which was present in 51.8% (*n* = 37) of CNBs. The others showed fibroadenomas in 28.6% (*n* = 20), phyllodes tumours in 15.7% (*n* = 11), and fibrocystic change in 2.9% (*n* = 2). The most commonly reported histologic subtype was benign (83.9%). 7.6% and 8.5% of the patients were of borderline and malignant histologic subtypes, respectively. The mean size of the mass was 4.57 cm, and the mean mitotic rate was 2.27 in 10 high-power fields (HPF). The type of breast surgery was mastectomy in 1.9%, breast-conserving surgery in 98.1%. Mastectomy was conducted in patients with malignant PT, characterized by a substantial tumor size occupying over half of the breast space, as it was impractical to achieve a clear margin in BCS. 39.4% of cases underwent second surgery. Totally, 52.8% of the cases had a negative surgical margin, and 47.2% of them had a positive surgical margin. All cases of malignant subtype had a negative surgical margin. In borderline and benign subtypes, 58.3% and 48.6% had a negative surgical margin. The demographics, imaging, and clinicopathologic characteristics of the study cohort are summarized in Table 1.

**Table 1:** Demographic and clinicopathologic features of the study cohort

Variables	Overall, (*n* = 318)	Recurrence, (*n* = 28)	No recurrence, (*n* = 290)	p value
Age, years				
Mean (SD)	36.97 (10.39)	36.10 (8.89)	37.05 (10.53)	0.643
Min, max	16, 86	21, 56	16, 86	
Age categories, n (%)				
≤ 36 years	142 (44.7)	14 (50)	128 (44.1)	0.558
> 36 years	176 (55.3)	14 (50)	162 (55.9)	
BMI				
Mean (SD)	25.41 (4.70)	25.15 (5.23)	25.44 (4.65)	0.815
Min, max	15.06, 39.03	15.2, 37.46	15.06, 39.03	
BMI categories, n (%)				
Non-obesity	265 (83.3)	24 (85.7)	241 (83.1)	0.723
obesity	53 (16.7)	4 (14.3)	49 (16.9)	
Breast feeding, n (%)	196 (61.6)	15 (53.6)	181 (62.4)	0.417
Oral contraceptive use, n (%)	70 (22)	12 (42.9)	58 (20)	0.008
Smoking, n (%)	17 (5.3)	5 (17.9)	12 (4.1)	0.002
Family history of breast cancer, n (%)	45 (14.2)	5 (17.9)	40 (13.8)	0.556
Side n (%)				
Left	148 (47.7)	9 (34.6)	139 (48.9)	0.162
Right	162 (52.3)	17 (65.4)	145 (51.1)	
Margins, n (%)				
Circumscribed	227 (71.4)	21 (75)	206 (71)	0.627
Micro lobulated	9 (2.8)	0 (0)	9 (3.1)	
Indistinct	82 (25.8)	7 (25)	75 (25.9)	
Echo pattern, n (%)				
Hypoechoic	308 (96.9)	24 (85.7)	284 (97.9)	< 0.001
Isoechoic	4 (1.3)	0 (0)	4 (1.4)	
Hyperechoic	6 (1.9)	4 (14.3)	2 (0.7)	
Surgical treatment n (%)				
Initial surgery				
Local excision	264 (83)	24 (85.7)	240 (82.8)	0.734
Wide excision	48 (15.1)	4 (14.3)	44 (15.2)	
Mastectomy	6 (1.9)	0	6 (2.1)	
Re-excision after local excision				
Yes	104 (39.4)	8 (33.3)	96 (40)	0.730
No	160 (60.6)	16 (66.7)	144 (60)	
Surgical margin n (%)				
Negative	168 (52.8)	12 (42.9)	156 (53.8)	0.268
Positive	150 (47.2)	16 (57.1)	134 (46.2)	
Size, cm				
Mean (SD)	4.57 (2.6)	6.09 (3.18)	4.42 (2.5)	0.004
Min, max	1,19	2, 13	1,19	
Size categories, n (%)				
≤ 4 cm	197 (61.9)	8 (28.6)	189 (65.2)	< 0.001
> 4 cm	121 (38.1)	20 (71.4)	101 (34.8)	
Histologic subtype n (%)				
benign	267 (83.9)	20 (71.4)	247 (85.1)	0.037
borderline	24 (7.6)	2 (7.1)	22 (7.6)	
malignant	27 (8.5)	6 (21.4)	21 (7.3)	
Mitotic Rate				
Mean (SD)	2.27 (3.74)	3.86 (4.03)	2.11 (3.68)	0.001
Min, max	0,30	0,13	0,30	
Mitotic Rate categories, n (%)				
≤ 2	241 (75.8)	14 (50)	227 (78.3)	0.001
> 2	77 (24.2)	14 (50)	63 (21.7)	
Heterogeneous stroma n (%)				
Absent	193 (60.7)	12 (42.9)	181 (62.4)	0.043
Present	125 (39.3)	16 (57.1)	109 (37.6)	
Stromal overgrowth n (%)				
Absent	216 (68.1)	10 (35.7)	206 (71.3)	< 0.001
Present	101 (31.9)	18 (64.3)	83 (28.7)	
Stromal cell atypia n (%)				
Absent	139 (44)	4 (14.3)	137 (46.9)	0.001
Present	177 (56)	24 (85.7)	153 (53.3)	

Age, size, and mitotic rate were stratified according to the median values

SD, standard deviation

From the overall study cohort, 28 (8.8%) reported local recurrence, and 290 (91.2%) did not. Distance recurrence was not found. Recurrence rate in benign, borderline, and malignant cases was 7.5%, 8.3%, and 22.2% respectively.

The relationship between tumor recurrence and tumor margin in all cases, as well as in benign and borderline cases separately was not significant (*p* > 0.05). In benign cases, the recurrence rate was 10.2% for positive margins and 4.6% for negative margins (*P* = 0.082). Similarly, in borderline cases, the recurrence rate was 10% for positive margins and 7.1% for negative margins (*p* = 0.803).

Patients who developed local recurrence experienced higher frequencies of oral contraceptive use, smoking, hyperechoic mass in sonography, malignant subtype, heterogeneous stroma, stromal overgrowth, and stromal cell atypia. Also, the mean size of the mass was larger, and the mean of mitotic rate was higher in patients who developed recurrence compared to the no recurrence group (*p* < 0.05). Cox regression analysis was performed to identify the predictors of local recurrence (Table 2). All possible risk factors were analyzed using the univariate regression model. Oral contraceptive use, smoking, hyperechoic mass in sonography, size > 4 cm, malignant subtype, mitotic rate > 2, presence of stromal overgrowth, and stromal cell atypia were the factors associated with recurrence. However, multivariate analysis demonstrated that oral contraceptive use (HR: 3.41; *p* = 0.002), smoking (HR: 3.17; *p* = 0.027), size > 4 cm (HR: 3.45; *p* = 0.005), presence of stromal overgrowth (HR: 2.81; *p* = 0.017), and presence of stromal cell atypia (HR: 3.65; *p* = 0.026) were the independent predictors of recurrence.

**Table 2:** Univariate and multivariate Cox regression models for the variables associated with urethral stricture recurrence

Variables	Univariate analysis		Multivariate analysis	
	HR (95% CI)	p value	HR (95% CI)	p value
Age > 36 years	0.80 (0.38, 1.67)	0.554	–	
Obesity	0.94 (0.66, 1.34)	0.742	–	
Breast feeding	0.70 (0.33, 1.48)	0.362	–	
Oral contraceptive use	2.86 (1.35, 6.06)	0.006	3.41 (1.57, 7.40)	0.002
Smoking	4.09 (1.55, 10.78)	0.004	3.17 (1.14, 8.82)	0.027
Family history of breast cancer	1.35 (0.51, 3.57)	0.535	–	
Hyperechoic mass	8.96 (3.10, 25.87)	< 0.001	3.14 (0.94, 10.49)	0.063
Surgical margin	1.70 (0.80, 3.63)	0.164	–	
Size > 4 cm	4.22 (1.86, 9.58)	0.001	3.45 (1.44, 8.27)	0.005
Histologic subtype				
benign	0.46 (0.20, 0.46)	0.066		
borderline	0.94 (0.22, 4.00)	0.943		
malignant	3.07 (1.24, 7.57)	0.015	0.94 (0.38, 3.05)	0.940
Mitotic Rate > 2	3.33 (1.59, 7.00)	0.001	0.82 (0.31, 2.16)	0.696
Heterogeneous stroma	2.11 (1.00, 4.47)	0.050	–	
Stromal overgrowth	4.07 (1.88, 8.82)	< 0.001	2.81 (1.20, 6.58)	0.017
Stromal cell atypia	5.15 (1.78, 14.89)	0.002	3.65 (1.16, 11.45)	0.026

The studied variables were categorized according to median

HR, hazard ratio; CI, confidence interval

**Fig. 1:**
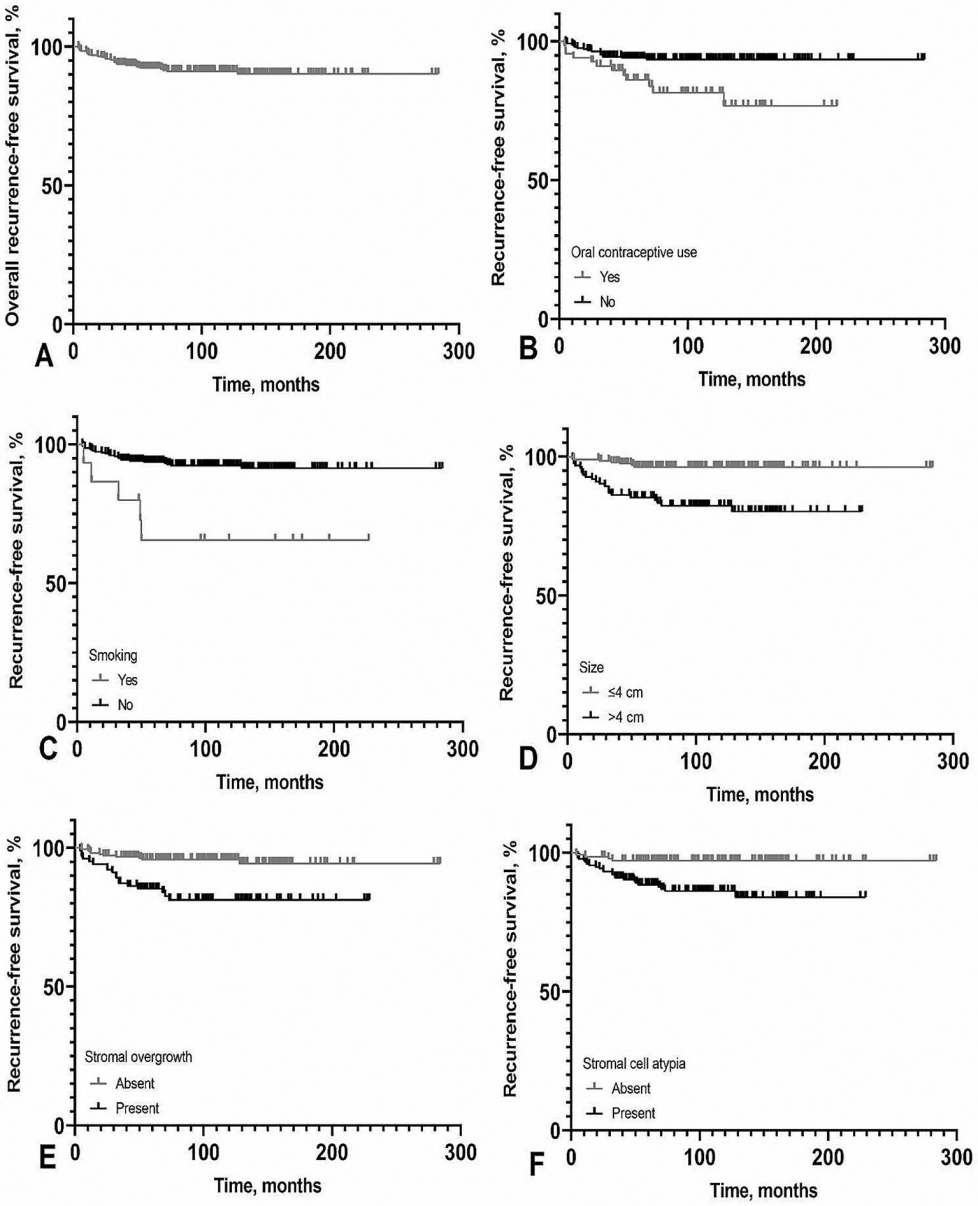
Kaplan-Meier survival plot of recurrence-free survival. Overall (**A**), and stratified by the presence or absence of risk factors: oral contraceptive use (**B**), smoking (**C**), size of mass (**D**), stromal overgrowth (**E**), and stromal cell atypia (**F**)

The recurrence-free survival overall and stratified by the presence or absence of risk factors are demonstrated in Fig. 1. The mean recurrence-free time was 259.99 months (95% CI: 251.43, 268.56 months). The mean time to recurrence was 32.07 months. The recurrence-free probability decreased with time; it was 97.2% after 1 year, 95.3% after 2 years, 92.1% after 3 years. The recurrence occurred in as early as 4 months and as late as 128 months postoperatively. Most of the recurrence (20 cases; 71.4%) occurred in the early postoperative 3 years. The mean recurrence-free survival times stratified by the presence or absence of risk factors are summarized in Table 3.

**Table 3:** Recurrence-free times according to the presence or absence of risk factors

	Mean stricture-free time, months (SD)	95% CI
Overall	259.99 (4.36)	251.43, 268.56
Oral contraceptive use		
Yes	180.05 (9.42)	161.57, 198.52
No	266.41 (4.11)	258.93, 275.06
Smoking		
Yes	203.51 (28.32)	147.99, 259
No	262.91 (4.27)	254.52, 271.29
Size		
≤ 4 cm	273.24 (3.72)	265.93, 280.54
> 4 cm	194.41 (7.07)	180.54, 208.29
Stromal overgrowth		
Absent	270.71 (4.19)	262,48, 278.94
Present	191.94 (7.92)	176.41, 207.47
Stromal cell atypia		
Absent	276.32 (3.78)	268.89, 283.74
Present	199.36 (5.70)	188.20, 210.58

CI, confidence interval; SD, standard deviation

**Fig. 2:**
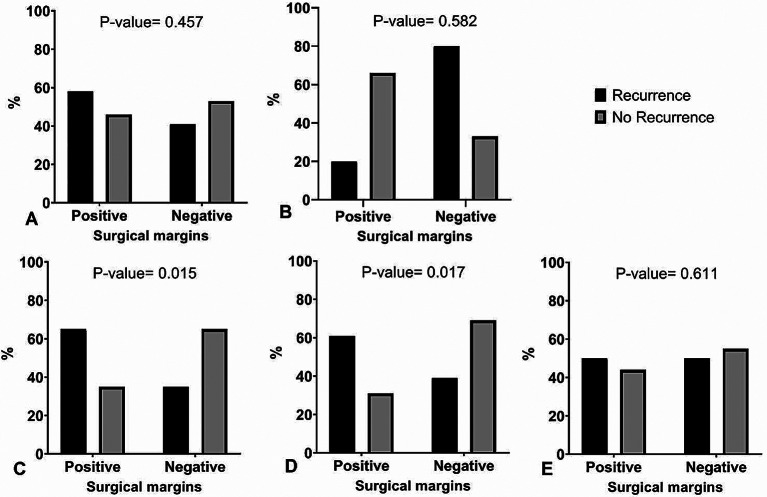
Bar charts of prevalence of recurrence according to surgical margin. stratified by the presence or absence of risk factors: oral contraceptive use (**A**), smoking (**B**), size of mass > 4 cm (**C**), stromal overgrowth (**D**), and stromal cell atypia (**E**)

**Fig. 3:**
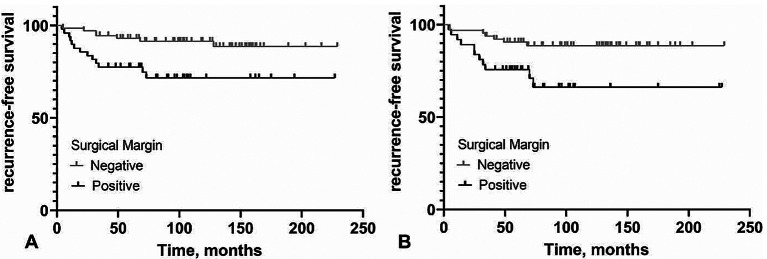
Kaplan-Meier survival plot of recurrence-free survival. stratified by the negative and positive surgical margin in mass size > 4 cm (**A**), and present of stromal overgrowth (**B**)

The results of this study showed that the negative surgical margin decreased the prevalence of recurrence in the size of the mass > 4 cm and the presence of stromal overgrowth significantly (*p* = 0.015, *p* = 0.017). The prevalence of recurrence according to surgical margin, stratified by the presence or absence of risk factors, is shown in Figs. 2 and 3, indicating the recurrence-free survival, stratified by the negative and positive surgical margin in mass size > 4 cm and the presence of stromal overgrowth.

## Discussion

Fibroepithelial lesions of the breast comprise a group of lesions ranging from fibroadenoma to malignant PT. The key difference in this group is the higher risk of recurrence in PT compared to fibroadenoma. NCCN guidelines were modified in 2021 and now recommend surgical excision without obtaining surgical margins for benign PT [24]. However, the latest published article suggests that having a free margin in the first surgery may be beneficial for reducing the recurrence rates [27]. This study aimed to identify the risk factors for PT recurrence and the effect of surgical margin on recurrence in patients with identified risk factors. Results showed a recurrence rate of 8.8%, and several factors, including oral contraceptive use, smoking, tumor size > 4 cm, malignant subtype, mitotic rate > 2, and the presence of stromal overgrowth and stromal cell atypia, were significantly associated with higher recurrence risk. Negative surgical margins may be beneficial in reducing the prevalence of recurrence in masses as large as 4 cm or with stromal overgrowth.

Our study demonstrated a recurrence rate of 8.8% at a median follow-up of 114.97 months. Recent studies have reported recurrence rates for PT ranging from 1.9 to 23.1% [20, 25, 28, 29]. The differences in recurrence rates across studies could be attributed to variations in the prevalence of malignant PT and other associated risk factors. Our study indicated that the recurrence rates for borderline and benign PT were near. Previous studies have shown that the recurrence of borderline phyllodes tumours is more similar to benign PT than malignant one [30–32]. Most recent studies have reported recurrence rates for benign phyllodes tumours ranging from 5 to 10%, which is consistent with our findings. A meta-analysis by Lu et al. reported a recurrence rate of 8% for benign phyllodes tumours [31], and Tan et al. analyzed 440 cases of benign phyllodes tumours and reported a recurrence rate of 10.9% [33]. However, Moldoveanu et al. and Moo et al. reported lower recurrence rates of 3.7% and 1.9%, respectively [20, 27].

The optimal management strategy for positive margins is still unclear. Of the 264 patients in our study with positive margins, just 39.4% (*n* = 104) underwent second surgery. Various studies have shown significant heterogeneity in margin management. Although older studies have recommended resection of tumors with wide margins [34–36], contemporary studies recommend a wait-and-watch approach [22, 30, 31, 37]. In some studies, re-surgery in tumors with positive margins has been reported to have no effect on recurrence [20]. However, considering the low rate of recurrence and potential complications, poor cosmetic outcomes, and additional costs associated with unnecessary interventions, margin revision may not be reasonable for both benign and borderline phyllodes tumours. Nevertheless, due to the possibility of tumor subtype change in subsequent recurrences, re-surgery may be helpful in some patients with identified recurrence risk factors.

Investigating the relationship between clinical and pathological characteristics of patients with PT and their risk of local recurrence is crucial for better personalizing surgical management. Previous studies have identified larger tumor size, presence of heterologous elements, high stromal cellularity, and high mitotic rate as risk factors for local recurrence. In our study, we found that oral contraceptive use for more than 6 months and smoking were also associated with disease recurrence, which has not been mentioned in previous studies. Additionally, we confirmed that larger tumor size, malignant subtype, high mitotic rate, stromal overgrowth, and stromal cell atypia were the risk factors for relapse, which is consistent with the findings of similar studies [27, 38]. Our study also revealed that tumors larger than 4 cm had a high risk of recurrence, and achieving a negative margin in this group of patients was significantly associated with a lower risk of recurrence. Notably, patients with stromal overgrowth who achieved a negative margin also had a significantly reduced risk of local recurrence.

Our study had several important limitations that should be acknowledged. First, the study population was small and limited to a single center, which may limit the generalizability of our findings to other populations or settings. Second, the study was retrospective in design, which means that data collection was based on past medical records, and there might be missing or incomplete data. This can introduce bias and limit the accuracy of the data collected. Although the follow-up interval was sufficient to capture most local recurrences, some may have been missed. Third, the radiology information of the patients was not performed by the radiologist of the unit and was extracted based on the report in the file; also, there was significant missing data in the radiology information. Fourth, the statistical methods used in the study might not have fully captured the complexity of the data. Therefore, the results of our study should be interpreted in the context of the available data and with caution. Prospective and multicenter studies are recommended for further investigation of the risk factors related to recurrence. Additionally, developing predictive models for recurrence risk could help identify high-risk patients who may benefit from a second surgery.

## Conclusions

The management of benign and borderline phyllodes tumors remains challenging, with local recurrences having a significant impact on patient quality of life and economic consequences. Our study found that achieving a negative margin in all patients did not significantly reduce the recurrence rate. Therefore, careful observation with serial ultrasound imaging and clinical physical examinations may be a reasonable alternative to correcting the margin in most patients with benign and borderline PT. However, achieving a negative margin may be effective in reducing recurrence in certain high-risk groups, such as those with tumors larger than 4 cm or with excessive stromal growth.

## Data Availability

Data is available from the corresponding author upon reasonable request via email.
